# Nutritional counselling in adults promoting adherence to the Mediterranean diet as adjuvant in the treatment of major depressive disorder (INDEPT): a randomized open controlled trial study protocol

**DOI:** 10.1186/s12888-023-04705-z

**Published:** 2023-04-04

**Authors:** Nuno Sousa-Santos, Mónica Fialho, Teresa Madeira, Cátia Clara, Sofia Veiga, Raquel Martins, Neuza Barros, Gabriela Santos, Osvaldo Santos, Carolina Almeida, Licínia Ganança, Rui C. Campos, José Camolas, Alda Pereira da Silva, Maria Pedro Sucena Guarino, Maria João Heitor

**Affiliations:** 1 Center for Innovative Care and Health Technology (ciTechcare), Instituto Politécnico, Leiria - R. de Santo André, Leiria, 2410 Portugal; 2 Instituto de Saúde Ambiental (ISAMB), Faculdade de Medicina, Universidade de Lisboa - Av, Lisboa, 1649-028 Portugal; 3 Laboratório Associado TERRA, Faculdade de Medicina, Universidade de Lisboa, Av. Professor Egas Moniz, Lisboa, 1649-028 Portugal; 4 Serviço de Psiquiatria e Saúde Mental, Centro Hospitalar de Leiria – Hospital de Santo André, R. de Santo André, Leiria, 2410-197 Portugal; 5 Departamento de Psiquiatria e Saúde Mental, Clínica Universitária de Psiquiatria e Psicologia Médica, Centro Hospitalar Universitário de Lisboa Norte, Lisbon, Portugal; 6 Comprehensive Health Research Center, Department of Psychology, School of Social Sciences, University of Évora, Évora, Portugal; 7 Centro Hospitalar Universitário Lisboa Norte, EPE, Hospital de Santa Maria, Av. Prof. Egas Moniz MB, Lisboa, 1649-028 Portugal; 8 Laboratório de Nutrição, Faculdade de Medicina, Universidade de Lisboa, Av. Prof. Egas Moniz, Edifício Egas Moniz, ala C, piso 2, Lisboa, 1649-028 Portugal; 9 Faculdade de Medicina da Universidade Católica Portuguesa - Estr. Octávio Pato, Rio de Mouro, Sintra, 2635-631 Portugal; 10 Clínica Universitária de Medicina Geral e Familiar, Faculdade Medicina Universidade de Lisboa, Av. Prof. Egas Moniz MB, Lisboa, 1649-028 Portugal; 11 Departamento de Psiquiatria e Saúde Mental, Hospital Beatriz Ângelo, Av. Carlos Teixeira 3, Loures, 2674-514 Portugal

**Keywords:** Randomized Controlled Trial, Major depressive disorder, Depression, Mediterranean Diet, Inflammation

## Abstract

**Background:**

Major Depressive Disorder (MDD) is a leading cause of disability worldwide. Approximately one-third of patients with MDD do not respond to treatment, and often exhibit elevated inflammation biomarkers, which are associated with worse prognosis. Previous research has linked healthier dietary patterns, such as the Mediterranean Diet (MedDiet), with a lower risk of MDD and symptoms of depression, potentially due to their anti-inflammatory properties. The aim of this study is to evaluate the effectiveness of a nutritional counselling intervention promoting MedDiet to alleviate symptoms of depression in adults recently diagnosed with MDD and presenting with elevated inflammation biomarkers.

**Methods:**

This study is a randomized controlled trial (RCT) that will recruit adults from outpatient clinics, between the ages of 18 and 70 years who have been diagnosed with MDD and are currently receiving treatment with the first prescribed antidepressant, and who exhibit elevated inflammation biomarkers (interleukin-6 and/or C-reactive protein). The control group will receive treatment-as-usual (TAU) only. The primary outcome of the study will be the change in symptoms of depression, as measured by the Beck Depression Inventory 2 (BDI-II), after 12 weeks of intervention. Data analysis will follow an intention-to-treat approach. Secondary outcomes will include changes in inflammation biomarkers, quality of life, adherence to the MedDiet, and cost-effectiveness of nutritional counselling. All outcomes will be assessed at baseline, after the 12-week intervention, and at 6- and 12-months post-baseline.

**Discussion:**

This study will be the first RCT to evaluate the effect of a nutritional intervention with anti-inflammatory properties, as an adjuvant in the treatment of MDD, in individuals diagnosed with MDD and elevated inflammation biomarkers. The results of this study may contribute to the development of more effective and personalized interventions for MDD patients with elevated inflammation biomarkers.

## Background

Major Depressive Disorder (MDD) is a global leading cause of disability, according to data from the Global Burden of Disease (2019) [1, 2]. It is a chronic condition, typically first diagnosed between mid-adolescence and mid-40s, usually characterized by alternating episodes of depression and remission, although some patients exhibit a persisting unremitting course [3]. In the individual’s life, MDD is associated with different adversities, namely a higher risk of cardiovascular diseases, physical comorbidities as well as associated with a reduced quality of life [4, 5]. When treated, these episodes tend to resolve within three to six months [3]. A significant challenge in treating MDD is the subset of patients who do not respond adequately to conventional treatment approaches, such as pharmacotherapy and psychotherapy [3]. Up to 60% of patients show insufficient response to the first antidepressant prescribed, and approximately 30% will fail to respond to multiple antidepressants, even with second-line medication, ultimately being diagnosed with treatment-resistant depression (TRD) [2, 3, 6].

Patients with non or partial to treatment response often show evidence of inflammatory dysregulation, including elevated levels of biomarkers such as Interleukin 6 (IL-6) and C-Reactive Protein (CRP), suggesting the involvement of inflammation pathways in the pathophysiology of MDD and resistance to pharmacological treatment [2]. Elevated pro-inflammatory cytokines have been linked to dysregulation of the hypothalamic-pituitary-adrenal axis, alterations in neurotransmitters metabolism, and decreased neuroplasticity, as indicated by reduced brain-derived neurotrophic factor levels [2]. These mechanisms have been implicated in the pathophysiology of MDD and pro-inflammatory cytokines have been proposed as negative predictors of treatment response [6–8]. Although not all patients show elevated levels of inflammation biomarkers, there is evidence that inflammation may play a role in some cases of MDD [9].

This hypothesis is supported by the high incidence of depression in patients with infections and autoimmune diseases, undergoing cytokine therapy, as well as in patients with Hepatitis C and cancer treated with interferon-alpha, who often experience elevated levels of pro-inflammatory cytokines and depression-like symptoms meeting the diagnostic criteria for MDD. In most cases, depression symptoms and inflammatory cytokines decrease shortly after treatment ends [9, 10]. Additionally, in patients with elevated inflammation biomarkers, Non-Steroidal Anti-Inflammatory Drugs (NSAIDs) are effective in treating MDD, albeit with the typical side effects of this drug class [8, 11].

The role of diet in the incidence and prognosis of chronic diseases, such as cardiovascular disease, type 2 diabetes mellitus, and certain types of cancer, is well-established [12]. In recent years, the evidence linking nutrition and mental well-being has been growing [13]. Diseases that show improvement with nutritional interventions promoting healthier diets often have an inflammatory nature, and the literature attributes the health benefits of these diets to their anti-inflammatory properties [14].

Although the relationship between diet and MDD as not been has extensively studied as other chronic diseases, the available evidence compiled in meta-analysis of observational studies have found an association between healthier dietary patterns, including high intake of fruit, vegetables, fish, and whole grains, and a lower risk of depression, also experimental data reported meta-analysis of randomized controlled trials (RCTs) found that dietary interventions aimed at promoting healthier diets resulted in a reduction in symptoms of depression [15–17].

Diet may act via several pathways that are implicated in mental health, including pathways related to oxidative stress, gut microbiota dysbiosis, mitochondrial dysfunction, and inflammation [18].

The relationship between inflammation and depression suggests that a dietary intervention with anti-inflammatory properties may have a positive effect in reducing pro-inflammatory biomarkers [18]. As such, patients diagnosed with MDD who have elevated pro-inflammatory markers may have a better response to standard psychiatric treatment if they adopt dietary interventions with anti-inflammatory effects [19].

One dietary pattern that has been extensively studied is the Mediterranean Diet (MedDiet). Greater adherence to this diet is associated with a reduced risk of chronic diseases and overall mortality [20]. Studies evaluating the MedDiet have shown a decrease in pro-inflammatory biomarkers, with some authors attributing these to the diet’s anti-inflammatory properties [21].

While the anti-inflammatory potential of the MedDiet has also been identified in some of its nutrients and foods, such as omega-3 fatty acids, extra virgin olive oil, fish, fruit, vegetables, and whole grain intake, the overall effects of the MedDiet dietary pattern have shown better results in reducing inflammatory biomarkers than the summed effects of its components (e.g., through supplementation) [22, 23].

The effects of a modified version of the MedDiet, adjusted for the Australian population, on MDD treatment has already been studied in a Randomized Clinical Trial (RCT) by Jacka and colleagues, with positive results. About a third (32.3%) of the participants in the modified MedDiet group achieved remission from MDD compared to 8% in the control group, however conclusions from this study are limited by its small sample size [24].

Growing evidence suggests that the elevation of inflammation may be a potential determinant of resistance to conventional treatments for MDD. Despite this, there is a lack of evidence from controlled clinical trials assessing the potential effect of the MedDiet in treating patients suffering from MDD. To the best of our knowledge, this will be the first RCT to promote adherence to the MedDiet and screen patients for their inflammatory profile before the intervention, to assess its impact on MDD symptoms. The hypothesis being tested in this trial is that promoting adherence to the MedDiet as an adjuvant to standard treatments for MDD can reduce symptoms of depression in patients with MDD and elevated inflammation biomarkers (IL-6 and CRP).

## Methods/design

### Objectives

The main objective of the trial is to determine the effectiveness of nutritional counselling promoting the MedDiet as an adjuvant treatment for MDD in reducing symptoms of depression in adults diagnosed with MDD and elevated inflammation biomarkers (CRP, IL-6).

Other specific objectives of the trial are:

1. To test the association between adherence to the MedDiet and changes in CRP and IL-6.
2. To test the association between changes in CRP and IL-6 and symptoms of depression.
3. To test the association between adherence to the MedDiet and alterations in symptoms of depression.
4. To identify the relationship between adherence to the MedDiet and changes in health-related quality of life.
5. To evaluate the economic cost-effectiveness of dietary counselling as an adjuvant treatment for MDD.

### Clinical trial register

The present study is registered at ClinicalTrials.gov Protocol Registration and Results System (PRS) under the number: NCT05745194. Any modifications to the study’s protocol will be submitted to the trial’s register prior to implementation.

### Study design

This is a 12-week, multicentre, randomized, parallel-group, open-label controlled trial. Participants will be randomly assigned in a 1:1 ratio to either: (a) the intervention group, which will receive six nutritional appointments with a registered nutritionist promoting adherence to the MedDiet, in addition to the usual treatment for MDD, or (b) the control group, which will only receive the usual treatment for MDD.

**Fig. 1:**
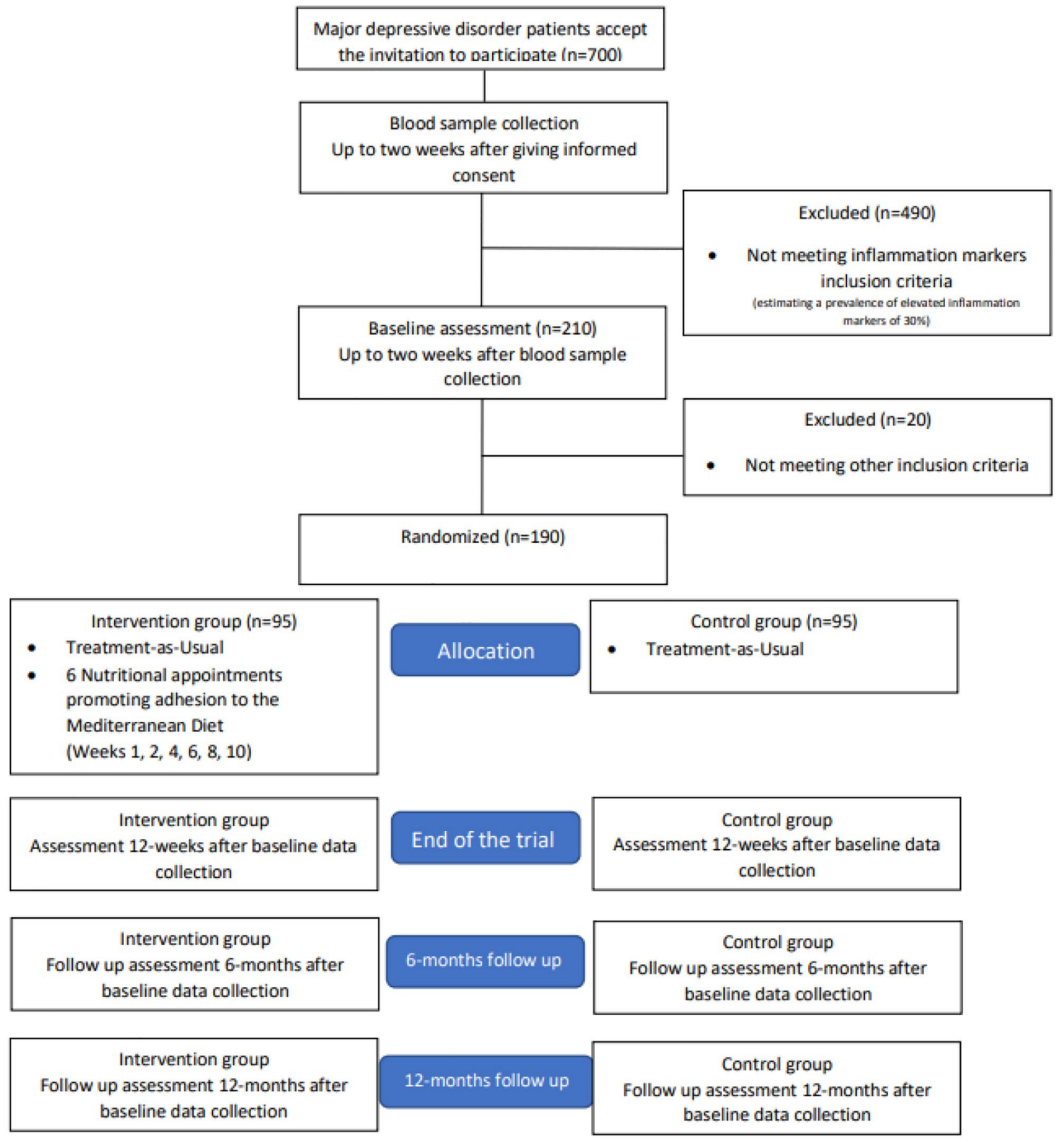
Recruitment and flowdiagram

Follow-up assessments will be conducted at 6 and 12 months after baseline assessment. More detailed information about the recruitment and allocation process is provided in Fig. 1.

This study protocol was developed by the Standard Protocol Items: Recommendations for Interventional Trials (SPIRIT) guidelines, and the results will be reported according to the Consolidated Standards of Reporting Trials (CONSORT) 2010 guidelines and their extension for non-pharmacologic treatments.

### Study participants, study setting and recruitment

The study will include adult (18–70 years old) outpatients diagnosed with MDD and having elevated levels of inflammation, as indicated by CRP ≥ 3 mg/L and/or IL-6 > 1.8pg/ml. The participants will be recruited from three Portuguese geographical areas: Lisbon, Loures/Odivelas, and Leiria, and will be recruited from hospitals and primary healthcare units. Three hospitals (Hospital de Santa Maria in Lisbon, Hospital Beatriz Ângelo in Loures, and Hospital de Santo André in Leiria) have already agreed to participate in the study, and two clusters of primary health care centres have expressed interest in collaborating. Additional recruitment health units are planned to be added during the trial.

Potential participants will be invited to enroll by their medical doctors at participating recruitment centres during regular medical appointments, including primary health care consultations and outpatient psychiatric consultations. After accepting the initial invitation, a researcher from the study team will provide a detailed explanation of the study and will obtain a signed informed consent from individuals who decide to participate. The collection of blood samples to measure the concentration of CRP and IL-6 will only take place after the informed consent is signed.

To be eligible for the trial, individuals must meet the following criteria:

#### Inclusion criteria

1. Aged between 18 and 70 years old;
2. Able to understand and provide informed consent;
3. Able to read and write;
4. Have a diagnosis of MDD (according to the International Classification of Diseases Version 10 F32.0; F32.1; F32.2 criteria).
5. Score on the Beck Depression Inventory-II scale (BDI-II) > 13;
6. Elevated biomarkers of inflammation (CRP ≥ 3 mg/l or IL-6 > 1.8pg/ml) [25];
7. Able to eat a MedDiet without impeditive physical limitations, allergies, or intolerances;
8. Treated with the first antidepressant prescribed for at least 4 weeks [26].

#### Exclusion criteria

1. Diagnosis of autoimmune diseases, thyroid dysfunction, or cancer;
2. Diagnosis of bipolar disorder, psychotic disorders, eating disorders or substance abuse disorders;
3. Reporting an infection 2 weeks before the blood sample collection;
4. Pregnancy or lactation;
5. Glucocorticoid medication;
6. Currently participating in another intervention targeting diet, physical exercise, or MDD treatment;

### Allocation and randomization

To ensure that the allocation of participants to the intervention and control arms is adequately done, a computer-generated list of random numbers will be used, stratified by centre, in variable blocks of sizes 2, 4, and 6. The treatment allocation will be concealed from the researchers and will be done using sequentially numbered, opaque, sealed envelopes. This will help prevent bias in the allocation process and ensure the validity of the study results. The researchers responsible for preparing the allocation sequence and the envelopes used in the randomization process will not have contact with the participants.

To maximize enrollment and retain participants, training and information about the trial will be provided to the medical doctors involved in recruitment through meetings, flyers, and emails. In addition, the consultations will be scheduled at times that are convenient for participants and will take place near their typical healthcare appointments. This will help minimize the burden of participation and promote retention to the study.

### Sample size

Considering a two-sided significance level of 5% and a power of 80% and using data from the trial published by Jacka et al. [24], the minimum sample size required to detect a difference of 20% points in the proportions of participants with a decrease in the severity of depressive symptoms, between control and intervention groups (8 and 28%, respectively), after 12 weeks, is 114 participants (57 per arm). Such difference corresponds to an odds ratio of 4.5, considered clinically relevant and smaller than the effect size derived from the SMILES trial [24]. Taking into consideration an attrition of 40% at the end of the intervention, the minimum sample size estimated is 190 (95 per arm). Estimating a prevalence of elevated inflammation in 30% of MDD patients and 10% of patients that don’t meet other inclusion criteria, the number of recruited participants estimated is 700 [2, 9].

### Intervention

Participants randomly allocated to the intervention group will attend six nutrition appointments with a duration between 30 and 60 min, in one of the participating centres of the trial, performed by a registered nutritionist in weeks 1, 2, 4, 6, 8 and 10. The intervention will promote adherence to the MedDiet without specific recommendations for weight change or calorie intake control.

Registered nutritionists involved in the intervention will participate in the baseline assessment but not in data collection at 12 weeks or at any of the follow-up assessments.

Both groups will maintain the TAU for MDD.

This trial will use a passive control group, with no active intervention apart from TAU.

All participants will have to be medicated with an antidepressant at the beginning of the trial. Changes in pharmacotherapy, including medicines and/or dosages, and additional treatments provided to participants (e.g., psychotherapy) will be documented and taken into consideration during statistical analysis.

### The mediterranean diet

Participants allocated to the intervention group will undergo six individual nutritional appointments, where they will receive individual counselling aiming to promote adherence to the MedDiet.

The MedDiet components promoted in nutritional appointments will follow a similar approach to the one used in the PREDIMED trial [27]. The nutritional counselling will not focus on calorie management or weight change and will be tailored to each participant’s dietary routines and preferences.

The intervention will contemplate the following recommendations:

#### Positive recommendations

1. Use of olive oil as main fat, both for cooking and dressing;
2. Consumption of two or more servings of vegetables daily (200 g of vegetables per serving);
3. Consumption of 2–3 servings of fresh fruits per day;
4. Intake of 3 or more servings of legumes per week;
5. Consumption of 3 or more servings of fish per week (100–150 g of fish per serving or 200 g of shellfish);
6. Consumption of 3 or more servings of nuts a week (30 g of nuts per serving);
7. Meat consumption mainly from poultry (without skin) or rabbit;
8. Homemade cooked meals with tomato, garlic, and onion, at least twice a week;

#### Negative recommendation

*Eliminate or drastically reduce the following foods*:

1. Cream, butter, and margarine;
2. Cold meats, pâtés, and duck meat;
3. Carbonated and/or sugared beverages;
4. Pastries and industrial baked products (cakes, doughnuts, or cookies);
5. Industrial deserts;
6. French fries and potato chips;
7. Pre-cooked cakes and sweets;
8. Alcohol should be limited to 300ml of wine per day (150 cc at meals for men and 100 cc for women, during meals).

Due to the recognized risk of interactions between alcohol and antidepressant medication [28], participants will be advised to completely avoid alcohol consumption. The recommendation to restrict the consumption of alcohol will only be given if participants report already including alcohol in their diets.

Training for nutritionists delivering the intervention will be provided prior to the beginning of recruitment, namely in MDD, trauma, therapeutic relationship, and in MedDiet. Additional training will be provided during the duration of the trial. This training will consider doubts and/or difficulties reported by the intervention team during the nutritional counselling as well as other topics considered relevant to promote adherence to MedDiet.

## Data collection and outcomes

### Main outcome: symptoms of depression

Symptoms of depression will be assessed with the self-administered Beck Depression Inventory-II (BDI-II) [28] scale, which is a 21 item self-report instrument for measuring the severity of depression in adults and adolescents. The total score is the sum of scores on the 21 items, ranging from 0 to 63. The adequate validity and reliability of this measure [29, 30] and its ability assessing depressive symptoms and monitoring the efficacy of treatment [30] makes it a valuable instrument for the current study. The Portuguese version of the BDI-II [30] will be administered. This version presents adequate internal consistency and convergent validity.

To measure changes in the main outcome, the score of the BDI-II will be used categorically, considering the cut-offs proposed by Beck et al. (1996) 0–13 minimal depression; 14–19 mild depression; 20–28 moderate depression; 29–63 severe depression [31].

The success measure will be a decrease in category in the severity of depressive symptoms.

The analysis of the score of the BDI-II will also be performed using the scale score as a continuous variable.

#### Time frame

Baseline, 12 weeks, 6 months, 12 months.

### Specific outcomes

#### Adherence to the MedDiet

Will be assessed by the Portuguese version of the 14-point Mediterranean Diet Adherence Screener [32, 33]. This instrument evaluates the intake of foods typical of MedDiet [33]. Changes between baseline and subsequent assessments will be analysed using the score of the scale as a continuous variable.

##### Time frame

Baseline, 12 weeks, 6 months, 12 months.

#### Changes in inflammation biomarkers (CRP and IL-6)

IL-6 and CRP concentrations in blood will be measured using an Atellica CH Analyzer, between baseline and other moments of assessment.

##### Time frame

Baseline, 12 weeks, 6 months, 12 months.

#### Health-related quality of life

Will be accessed with the World Health Organization Quality of Life – Brief (WHOQOL-BREF), a 26-item short version of the more extensive WHOQOL-100 [34]. Changes in Health-related quality of life will be measured, between baseline and other moments of assessment using the score of the WHOQOL-BREF as a continuous variable.

##### Time frame

Baseline, 12 weeks, 6 months, 12 months.

#### Economic costs associated with MDD

To conduct the economic cost analysis of the intervention self-reported information will be obtained for the following variables: health care resource use (medicines, appointments, hospital admissions and other unspecified healthcare services) and employment status (occupation, current employment situation, workplace absenteeism and presenteeism). The estimation of costs associated with MDD will be calculated for healthcare resource use and the indirect costs to patients.

##### Time frame

Baseline, 12 weeks, 6 months, 12 months.

## Co-variables

### Sociodemographic variables

Sex, age, level of education, household composition, professional occupation, and nationality.

#### Time frame

Baseline.

## Medical history

Self-reported medical diagnosis, medication, self-reported allergies and food intolerances.

### Time frame

Baseline.

## Thyroid function

Thyroid-stimulating hormone (TSH), Free Thyroxine (fT4) will be measured using Atellica IM Analyzer.

### Time frame

Baseline.

## Lifestyle variables

### Physical activity

Physical activity will be assessed with the self-administered Portuguese version of the International Physical Activity Questionnaire – Short Form (IPAC-SF) [35].

#### Time frame

Baseline, 12 weeks, 6 months, 12 months.

## Smoking and drinking habits

Smoking and drinking habits will be assed using a customized survey instrument.

### Time frame

Baseline, 12 weeks, 6 months, 12 months.

### Anthropometric measurements

#### Weight, height, Body Mass Index.

Anthropometric measurements will be performed at the end of data collection by a professional not involved in the intervention. Weight will be measured using a scale DIGITAL SECA 813 and height will be assessed with a stadiometer SECA 213.

##### Time frame

Baseline, 12 weeks, 6 months, 12 months.

## Liver function

Alanine aminotransferase (ALT), Aspartate transaminase (AST), Gamaglutamil Transpeptidase (GGT) will be measured using Atellica CH Analyzer.

### Time frame

Baseline, 12 weeks, 6 months, 12 months.

## Blood cells

Hemogram and leukogram will be measured using Coulter System DxH 800.

### Time frame

Baseline, 12 weeks, 6 months, 12 months.

## Glucose metabolism

Hemoglobin A1C (HbA1C) will be measure using Bio-Rad D-100™.

### Time frame

Baseline, 12 weeks, 6 months, 12 months.

Detailed information regarding blood work analyses is described in Table 1.

**Table 1:** Information about blood work analysis

Biomarker	Test name	Product Name	Systems	Measurement interval
Hemogram	Hemogram	Coulter	System DxH 800 Coulter	NA
Leukogram	Leukogram	Coulter	System DxH 800 Coulter	NA
hs-CRP	wrCRP	Atellica CH wrCRP	Atellica CH Analyzer	0,050–15,600 mg/dl (0,50–156,00 mg/l)
IL-6	IL-6	Atellica IM IL6	Atellica IM Analyzer	2,7–5500,0 pg/ml
ALT	ALT	Atellica CH ALT	Atellica CH Analyzer	7–1100 U/l
AST	AST	Atellica CH AST	Atellica CH Analyzer	8–1000 U/l
GGT	GGT	Atellica CH GGT	Atellica CH Analyzer	7–1200 U/l
FT4	FT4	Atellica IM FT4	Atellica IM Analyzer	0,1–12,0 ng/dl (1,3–154,8 pmol/l)
TSH	TSH3‑UL	Atellica IM TSH3‑UL	Atellica IM Analyzer	0,008–150,000 µIU/ml (mIU/l)
HbA1c	HbA1c	D-100TM HbA1c	Bio-Rad D-100™	NA

### Consent, confidentiality and data access

Only individuals who have provided written informed consent will be enrolled in the study. Participants will be informed of their right to withdraw from the study at any time without providing justification and will be assured that their decision will not have any negative impact on the provision of their normal healthcare services. Only their names and other sensitive identifying information will be stored in a physical format to protect participant privacy. Participants will be granted the right to access their data upon request.

### Discontinuation of participation and missing data

Participant’ discontinuation is expected to occur in the following scenarios: hospitalization during the trial, development of conditions that restrict adherence to the MedDiet, diagnosis of a disease that is incompatible with the intervention, or initiation of another nutritional intervention. Baseline data from participants who are excluded after baseline assessment will be included in the sample characterisation.

Missing data from participants that don’t complete all the steps planned in the trial will be analysed following an intention-to-treat (ITT) approach.

Motives of participant exclusion or abandonment will be documented and reported if the information is available.

Due to the nature of the intervention, the promoting adherence to the MedDiet, negative reactions are not expected.

### Data management

All data collected will be de-identified and aggregated prior to analysis.

To ensure the quality of data from printed questionnaires, data entry and data quality validation will be performed by two researchers.

Secure systems with encryption and password protection will be used to store databases, and strong, unique passwords will be assigned. To minimize the risk of data loss, periodic backups will be performed and stored in separate information systems. Access to data prior to de-identification and aggregation will be limited to the research team, unless participants provide additional informed consent for individual access.

### Statistical analysis

Data analysis and reporting will be performed in accordance with CONSORT guidelines.

Analysis of the primary outcome will include all randomized participants, following an ITT approach.

Baseline characteristics, by group, will be reported using descriptive statistics and compared by χ2 and t-test for independent samples (or equivalent non-parametric test). To evaluate the effectiveness of the intervention, the proportion of participants with a decrease in depression severity symptoms category according to the BDI-II (main outcome) will be compared between control and intervention groups using generalised linear mixed models.

Further adjustment for relevant covariates, as supportive analysis, will be made. Secondary analyses include between-group differences over time in symptoms of depression, considering the BDI-II as a continuous variable. Regarding specific objectives, the analysis of symptoms of depression (BDI-II scale score) given the levels of CRP and IL-6 will be conducted using linear mixed models, adjusted for age, BMI, hemoglobin HbA1C, medication, physical activity, smoking and drinking habits and sex. The same method will be applied to test the effect of adherence to MedDiet on the levels of the biomarkers (CRP and IL-6) and in health-related quality of life. The association between adherence to MedDiet and changes in symptoms of depression will also be investigated, using, for this purpose, generalized linear mixed models. The economic cost-effectiveness of dietary counselling as an adjuvant treatment for MDD, compared to the usual treatment, will be presented as an incremental cost-effectiveness ratio, given the differences in the total costs of each treatment and their health effects (i.e., the proportion of patients with improvement in symptoms of depression after 12 weeks of intervention). For each outcome, results for either intervention and control group, and estimates of effect size with a 95% confidence interval will be reported. All analyses will be performed assuming a significance level of 5%.

### Benefits, risks and compensation of participants

Participants assigned to the intervention group will receive personalized nutritional guidance from a registered nutritionist who will promote adherance to the MedDiet, which has been shown to have health benefits in several chronic diseases that are commonly co-occurring with MDD. At the conclusion of the study, results and findings will be shared with all participants. If the study hypothesis is confirmed, individuals in the control group will be provided with information on how to improve their adherence to the MedDiet. Aside from the standard risks associated with blood sample collection, no additional risks are expected with the proposed intervention. No financial or gift compensation will be provided.

### Discontinuation of participation and missing data

Participants discontinuation is expected to occur in the following scenarios: hospitalization during the trial, development of conditions that forbid adherence to the MedDiet, diagnosis of a disease that is incompatible with the intervention, or initiation of another nutritional intervention. Baseline data from participants who are excluded after baseline assessment will be included in the sample characterisation.

Missing data from participants that don’t complete all the steps planned in the trial will be analysed following an ITT approach.

Motives of participant exclusion or abandonment will be documented and reported if the information is available.

Due to the nature of the intervention, the promoting adherence to the MedDiet, negative reactions are not expected.

### Dissemination policy

The results of the study will be disseminated to interested parties, including the professionals involved in the study’s design and implementation, the scientific community, mental health professionals, and the public at large. A seminar to present the results and conclusions will be held at the end of the data analysis, with additional presentations taking place at the recruitment centres. The findings of the study will be shared with the scientific community through (1) presentations at conferences and meetings related to nutrition, medical nutrition, psychiatry, psychology, and epidemiology; (2) publication in scientific peer-reviewed journals; and (3) a final report, which is a requirement of the funding entity for the study.

## Discussion

Even though treatments are available, about one-third of patients with MDD don’t find sufficient relief of symptoms even after several treatment approaches [2]. Increased inflammation biomarkers have been associated with a higher risk of developing MDD and diminished efficacy of conventional treatments [2, 3].

This study aims to understand if promoting adherence to the MedDiet, as an adjuvant strategy in the treatment of MDD, is effective to decrease symptoms of depression in patients diagnosed with MDD with high levels of inflammation biomarkers, namely elevation of CRP and/or IL-6 at baseline.

### Strengths

This study will be the first RCT to evaluate the effect of a nutritional intervention with anti-inflammatory properties, as an adjuvant in the treatment of MDD, in individuals diagnosed with MDD and elevated CRP and/or IL-6 biomarkers. The selection of individuals with elevated inflammation biomarkers, will potentially target patients that might benefit more from the proposed intervention.

As participants will be recruited during their routine medical appointments and the intervention will take place near recruitment centres, the trial conditions will closely resemble routine care, reducing the potential for bias. The use of a passive control group that will only receive TAU will allow for an evaluation of the impact of the proposed intervention compared to standard care. The cost-effectiveness analysis will provide information on the scalability of the proposed treatment.

### Limitations

There is a potential challenge in recruiting a sufficient sample size for this study, as the condition of having elevated CRP and IL-6 levels will increase the number of participants needed to be invited. Similar difficulties have been identified in a previous study [24]. To address this, participant recruitment and data collection will happen simultaneously in three hospitals, with more recruitment centres planned to be added as the trial progresses.

Due to the nature of the study, which involves promoting adherence to a specific diet, it will not be possible to blind the participants or researchers delivering the intervention to allocation groups. To minimize the resulting bias, the researchers responsible for statistical analysis will be blinded to allocation groups. Using the PREDIMED-MEDAS questionnaire to assess MedDiet adherence and measuring inflammation biomarkers at each assessment point will help to identify any associations between alterations in diet adherence, inflammation biomarkers, and changes in depression symptoms.

The study design involves a passive control group that will receive no active intervention besides TAU. This approach eliminates the risk of comparing two effective interventions, but increases the risk that differences found between groups could be due to differences in intervention intensity between groups.

## Conclusion

This study will contribute to the understanding of the role of inflammation and nutrition in the treatment of MDD, in a group of patients with a lower remission rate with usual treatments, with potential gains in terms of improving health and reducing healthcare costs.

## Data Availability

Data available at clinicaltrials.gov (NCT05745194).

## List of abbreviations

BDI-II: Beck Depression Inventory II
CRP: C-Reactive Protein
IL-6: Interleukin 6
MDD: Major Depressive Disorder
MedDiet: Mediterranean Diet
RCT: Randomized clinical trial
TAU: Treatment as usual
TRD: Treatment Resistant Depression
